# TWEAK/Fn14 disrupts Th17/Treg balance and aggravates conjunctivitis by inhibiting the Nrf2/HO-1 pathway in allergic conjunctivitis mice

**DOI:** 10.1186/s10020-024-01004-5

**Published:** 2024-11-26

**Authors:** Yang Yang, Yuezhi Zhang, Jingfan Fu, Xiaolong Yin

**Affiliations:** Department of Ophthalmic Center, The Second Affiliated Hospital of Nanchang University, No. 1 Minde Road, Nanchang, 330006 Jiangxi China

**Keywords:** Allergic conjunctivitis, TWEAK/Fn14, Nrf2/HO-1 pathway, Th17/Treg

## Abstract

**Background:**

Allergic conjunctivitis (AC) affects people’s daily life and work, especially the health of children. Although there are few relevant studies, Th17/Treg imbalance plays an important role in AC development. The aim of this study was to elucidate the effect of TWEAK/Fn14 on AC and Th17/Treg balance.

**Methods:**

Ovalbumin induced AC mouse model was utilized to observe the mechanism of TWEAK/Fn14 in vivo. Conjunctivitis was evaluated by hematoxylin-eosin staining, toluidine blue staining and AC clinical score. Flow cytometry was used to measure Th17 and Treg cell ratios. The level of Th17/Treg balance related factors and Nrf2/HO-1 signal was detected by ELISA, WB, qRT-PCR and immunohistochemistry.

**Results:**

In the AC state, disruption of Th17/Treg cell balance, increased TWEAK/Fn14 signaling level and conjunctival inflammation were observed. After TWEAK knockdown, Th17 cell differentiation was inhibited, Treg cell differentiation was promoted, and AC symptoms were alleviated in AC mice. Moreover, TWEAK knockdown caused an enhancement of the Nrf2/HO-1 signaling pathway in the AC models. Treatment with Nrf2 inhibitor reversed these changes induced by TWEAK knockdown. Therefore, TWEAK/Fn14 regulated the Nrf2/HO-1 pathway to affect Th17/Treg cell balance and conjunctivitis in AC mouse models.

**Conclusion:**

In summary, TWEAK/Fn14 caused Th17/Treg imbalance by inhibiting Nrf2/HO-1 pathway, which might be one potential mechanism of the exacerbation of AC.

**Supplementary Information:**

The online version contains supplementary material available at 10.1186/s10020-024-01004-5.

## Background

Allergic conjunctivitis (AC) is a prevalent ocular condition, affecting 15-40% of individuals, with children being the most susceptible demographic (Patel et al. 2017). AC is primarily triggered by allergens such as pollen, dust, and mites (La Rosa et al. 2013). Clinical manifestations of AC include itching, redness, tearing, dryness with a burning sensation, periorbital edema with increased secretions, and in severe cases, vision impairment (Villegas and Benitez-Del-Castillo 2021).

CD4^+^ T cells play a crucial role in the regulation of conjunctival inflammation (Magaña et al. 2015; Shaker and Salcone 2016). The activated CD4 T cells exert their effects by differentiating into distinct subsets with varying functions, including T helper 17 (Th17) cells and regulatory T (Treg) cells (Lee 2018). The Th17/Treg balance is a crucial factor in the pathogenesis of autoimmunity. Th17 cells promote inflammation through the secretion of IL-17 A, IL-17 F, and IL-22 cytokines, whereas Treg cells suppress inflammation primarily through the release of IL-10, TGF-β, and other cytokines (Savage et al. 2020). Research has demonstrated that the transcription factor RORγt, which governs Th17 cell differentiation, is upregulated in AC mice, while the transcription factor foxp3, responsible for Treg cell differentiation, is downregulated (Chen et al. 2019). The dysregulation of Th17/Treg balance may significantly contribute to the initiation and progression of AC, although there is a paucity of relevant investigations on this topic.

Nrf2/HO-1 signaling pathway, composed of Nuclear factor erythroid-2 related factor 2 (Nrf2) and Heme oxygenase-1 (HO-1), exhibits anti-inflammatory and antioxidant functions (Zhang et al. 2021a). Studies have corroborated that the activated Nrf2/HO-1 signaling pathway inhibits Th17 cell differentiation and promotes Treg cell differentiation, thus playing an anti-inflammatory role (Chen et al. 2017; Van Nguyen et al. 2020). In the AC state, whether the Nrf2/HO-1 signaling pathway has the same performance attracts our attention.

Tumor necrosis factor-like weak inducer of apoptosis (TWEAK), a member of the tumor necrosis factor (TNF) ligand family, has the ability to induce apoptosis (Chicheportiche et al. 1997). Fibroblast growth factor-inducible 14 (Fn14), a type I transmembrane receptor belonging to the TNF receptor family, is a receptor of TWEAK (Wiley et al. 2001; Winkles 2008). TWEAK regulates many biological processes through Fn14, including cell proliferation, angiogenesis, and inflammation (Burkly et al. 2011). Moreover, TWEAK has been found to elevate the levels of pro-inflammatory cytokines and is implicated in the development of various inflammatory and autoimmune disorders (Gupta et al. 2021; Kamata et al. 2006). Despite this, the role of TWEAK/Fn14 in AC remains unexplored. TWEAK has also been verified to play a role in promoting the differentiation of T cells into the Th17 phenotype (Park et al. 2012). Furthermore, in a mouse model of acute lung injury, heightened expression of Fn14 has been shown to trigger oxidative stress and inflammation, leading to the inhibition of the Nrf2/HO-1 signaling pathway (Guan et al. 2022). In summary, we hypothesize that TWEAK/Fn14 promotes dysregulation of Th17/Treg balance by inhibiting the Nrf2/HO-1 signaling pathway, thereby aggravating AC progression.

The objective of this research was to investigate the involvement of the TWEAK/Fn14 signaling axis and Nrf2/HO-1 signaling pathway in AC by constructing AC mouse models. The findings implied a disruption in the Th17/Treg balance of these models. Besides, it was observed that TWEAK/Fn14 facilitated Th17 cell differentiation by suppressing the Nrf2/HO-1 signaling pathway, resulting in exacerbated conjunctivitis in AC mice. This study highlights a promising potential therapeutic target for the management of AC.

## Materials and methods

### Animals

The Balb/c male mice (aged 8 weeks, weight 20–25 g) (Spiff Biotechnology Co., LTD, Beijing, China) were housed in the breeding environment of the Specific Pathogen Free grade standard, keeping constant temperature (23–27 °C) and constant humidity (40–70%). In addition, the mice were given daily adequate food and water and treated alternately according to a cycle of 12 h light and 12 h darkness. The experiment was carried out after one week of adaptive feeding. All protocols were authorized by the Ethics Committee of The Second Affiliated Hospital of Nanchang University.

### AC mouse model

Balb/c mice were randomly divided into Control and AC groups. On days 1 and 8, mice in the AC group received an intraperitoneal injection of 200 µL saline containing 10 µg ovalbumin (OVA) (Sigma Aldrich, St. Louis, MO, USA) and 200 µL aluminum hydroxide adjuvant (Yuanye Biotechnology Co., LTD, Shanghai, China) to promote drug sensitization. To establish a model of antigen-induced conjunctivitis, 20 µL of normal saline containing 200 µg OVA was dropped into the eyes of AC group mice once daily between days 15 and 21. The mice in the Control group were treated with intraperitoneal injection and eye drops of equal dose of normal saline at the same time point in the same way. Twenty-four hours after the last eye drops of OVA (day 22), the mice in each group were sacrificed in sate of anesthesia and their eyeballs were enucleated to obtain their conjunctival tissues and spleen. One eye of each animal was taken for histological examination, and the other examinations were performed using the contralateral eye.

### Evaluation of clinical symptoms

The 20 min after the last drop of liquid was drip into eyes, the clinical evaluation of AC was conducted in a masked fashion by two independent well-trained observers. Clinical scoring consisted of five parameters: lid swelling, conjunctival chemosis, conjunctival redness, corneal injury and secretion. Each limitation was scored on a scale from 0 (absence of signs) to 4 (maximal), and the sum of the scores for the five parameters yielded the total score for each animal.

### Adeno-associated virus (AAV) transfection

To investigate the effect of TWEAK in AC mouse models, AAV-shTWEAK and AAV-shNC (Gemma Biotechnology Co., LTD, Suzhou, Jiangsu, China) were constructed. The mice were randomly divided into 4 groups. Specifically, 5 µl AAV-TWEAK and AAV-shNC was respectively injected into the conjunctival site of the AC + AAV-shTWEAK group and the AC + AAV-shNC group on the 9th day of modeling. In the same way, mice in CON + AAV-shNC group and CON + AAV-shTWEAK group were respectively injected into conjunctival with AAV-shNC and AAV-shTWEAK, but OVA induction was not performed. To explore the role of Nrf2 pathway/HO-1 in TWEAK regulating the Th17/Treg balance, AC mice were treated with subconjunctiva injection of Nrf2 inhibitor ML-385 (MedChemExpress, Shanghai, China) after injection of AAV-shNC or AAV-shTWEAK in the conjunctiva.

### Enzyme-linked immunosorbent assay (ELISA)

The levels of IL-17 A, IL-17 F, IL-22, TGF-β, and IL-10 in the spleen were assessed using the ELISA kits according to the instructions. The ELISA kits were used as follows: Mouse IL-17 A ELISA Kit (E-EL-M0047c, Elabscience, Wuhan, Hubei, China), Mouse IL-17 F ELISA kit (ab204522, Abcam, Cambridge, MA, USA), Mouse IL-22 ELISA kit (E-EL-M2446c, Elabscience), Mouse IL-10 ELISA kit (E-EL-M0046c, Elabscience) and Mouse TGF-β ELISA kit (JL13959, Jianglaibio, Shanghai, China).

### Quantitative real-time fluorescence PCR (qRT-PCR)

The qRT-PCR assay was utilized to measure the mRNA levels of TWEAK, Fn14, Nrf2, and HO-1 in conjunctival tissue. In brief, total RNA was extracted by TRIzol reagent (Invitrogen, Waltham, MA, USA). The obtained total RNA was reversely transcribed into cDNA with PrimeScript RT reagent Kit (Takara, Tokyo, Japan). The mRNA expression level was detected by TB Green QuantiTect RT-PCR kit (Takara) with GAPDH as the internal control. After each reaction was repeated three times, the data were analyzed using 2^−∆∆CT^ method. The primers used in qRT-PCR were listed in Table 1.

**Table 1:** Primers of qRT-PCR

Gene	Forward sequence (5’-3’)	Reverse sequence (5’-3’)
TWEAK (Human)	GAGGGGAAGGCTGTCTACCT	GAACCTGGAAGAGTCCGAAGTA
TWEAK (Mouse)	CCGCCAGATTGGGGAATTTAC	AGTCCAAAGTAGGTTAGGAAGGG
Nrf2 (Human)	TCAGCGACGGAAAGAGTATGA	CCACTGGTTTCTGACTGGATGT
Nrf2 (Mouse)	CTTTAGTCAGCGACAGAAGGAC	AGGCATCTTGTTTGGGAATGTG
Fn14 (Human)	GAGGCAAGACCGAAGTAAACTAC	CCGAACTGGTTACACGGGAA
Fn14 (Mouse)	GTGTTGGGATTCGGCTTGGT	GTCCATGCACTTGTCGAGGTC
HO-1 (Human)	AAGACTGCGTTCCTGCTCAAC	AAAGCCCTACAGCAACTGTCG
HO-1 (Mouse)	AGGTACACATCCAAGCCGAGA	CATCACCAGCTTAAAGCCTTCT
GAPDH (Human/ Mouse)	GCACCGTCAAGGCTGAGAAC	TGGTGAAGACGCCAGTGGA

### Hematoxylin eosin (HE) staining

The conjunctival tissue fixed with 4% paraformaldehyde was dehydrated with ethanol and xylene, followed by paraffin embedding. Then, the intact tissue was cut into wax slices with a thickness of 4 μm. The dried wax slices were dewaxed. After rinsing with distilled water, sections were successively stained with hematoxylin (Servicebio, Wuhan, Hubei, China) for 5 min and eosin (Servicebio) for 5 s. After being washed with distilled water again, the slices were dehydrated and sealed. Subsequently, the staining of the conjunctival tissue was observed with a microscope.

### Toluidine Blue (TB) staining

Mast cells in the conjunctival tissue were detected by TB staining. The tissue sections were dewaxed using dewaxing solution (Servicebio) and ethanol. After rinsing with tap water, sections were treated with TB dye liquor (Servicebio) for 2–5 min and then slightly differentiated by 0.1% glacial acetic acid. Subsequently, when a suitable degree of differentiation was observed under the microscope, the sections were washed to terminate the reaction. After drying, the slices were sequentially transparent with xylene and sealed with neutral gum. Finally, the staining was observed with a microscope, with light blue background and purplish blue mast cell granules.

### Immunohistochemical (IHC)

After deparaffinization and rehydration, tissue slides were treated with 100 µL endogenous peroxidase blockers for 10 min. Then, the slides were incubated with primary antibody against RORγt (14-6981-82, 1:200; ThermoFisher, Waltham, MA, USA), FoxP3 (BA2032-1, 1:200; Boster, Pleasanton, CA, USA), TWEAK (BM4635, 1:200; Boster), Fn14 (GTX85216; 1:200, Genetex, Alton, CA, USA), Nrf2 (PA5-27882; 1:200, ThermoFisher) and HO-1 (PA5-77833, 1:200; ThermoFisher) overnight at 4 °C. The tissue slides were treated with appropriate amount of enzyme-labeled goat anti-mouse IgG polymer (PV-6000, 1:1000; Zhongshan Jinqiao Biotechnology Co., LTD, Beijing, China) at 37 °C for 20 min. Sections were stained with a DAB kit (Servicebio) followed by hematoxylin. Finally, the protein staining was observed under microscope.

### Flow cytometry

The proportion of Th17 and Treg cells was observed by flow cytometry. Briefly, the obtained spleen cells were prepared into single-cell suspension. After culture for 24 h, centrifuge it in 1200 rpm for 10 min and discard the upper clearance. Subsequently, the resulting mixture was incubated with 0.2 µg/100µL FITC anti-mouse CD4 Antibody (100405, BioLegend, San Diego, CA, USA), PE anti-mouse CD25 Antibody (113703, BioLegend), and Alexa Fluor^®^ 647 anti-mouse FOXP3 Recombinant Antibody (118905, BioLegend) to detect the Treg cells at 4℃ overnight in the dark. The Th17 cells were detected by incubating the mixture samples with 0.2 µg/100µL FITC anti-mouse CD4 Antibody (100405, BioLegend) and APC anti-mouse IL-17 A Antibody (506915, BioLegend) under the same conditions. After being resuspended with the membrane breaking buffer (Invitrogen) and the flow dyeing buffer (Invitrogen) successively away from light, the samples were analyzed using flow cytometer (Agilent, Santa Clara, CA, USA).

### Western blotting (WB)

RIPA lysis buffer (ThermoFisher) was utilized to lyse the tissue cells. After being detected with a bicinchoninic acid (BCA) kit (Biosharp, Guangzhou, Guangdong, China), the obtained proteins were separated by SDS-PAGE before transferred onto polyvinylidene fluoride (PVDF) membranes (Millipore, Billerica, MA, USA). Membranes were blocked by BSA (5%) for 1 h at room temperature and then cultured with primary antibodies against RORγt (ab207082, 1:1000; Abcam), FoxP3 (ab20034, 1:1000; Abcam), TWEAK (BM4635, 1:1000; Boster), Fn14 (A11813, 1:1000; Boster), Nrf2 (ab62352, 1:1000; Abcam), HO-1 (ab68477, 1:1000; Abcam) and GAPDH (ab8245, 1:1000; Abcam) at 4 °C overnight. After washing five times with TBST, the membranes were incubated with horseradish peroxidase (HRP)-conjugated goat anti-rabbit secondary antibody (ab6721, 1:5000; Abcam) and HRP-conjugated goat anti-mouse secondary antibody (ab6789, 1:5000; Abcam) for 2 h at 37 °C. Finally, we detected the brightness of protein bands using enhanced chemiluminescence (ECL) (Bio-rad, Shanghai, China).

### Statistical analysis

Each assay was performed for 3 times. Data were analyzed by GraphPad Prism 6.0 (La Jolla, CA, USA) and expressed as mean ± standard deviation. Two-tailed Student’s *t* test were used for comparing two variables. One-way ANOVA test was used for multiple variable comparison. *P* < 0.05 was considered as a significant difference.

## Results

### AC affected Th17/Treg cell differentiation and TWEAK/Fn14 signaling level

**Fig. 1:**
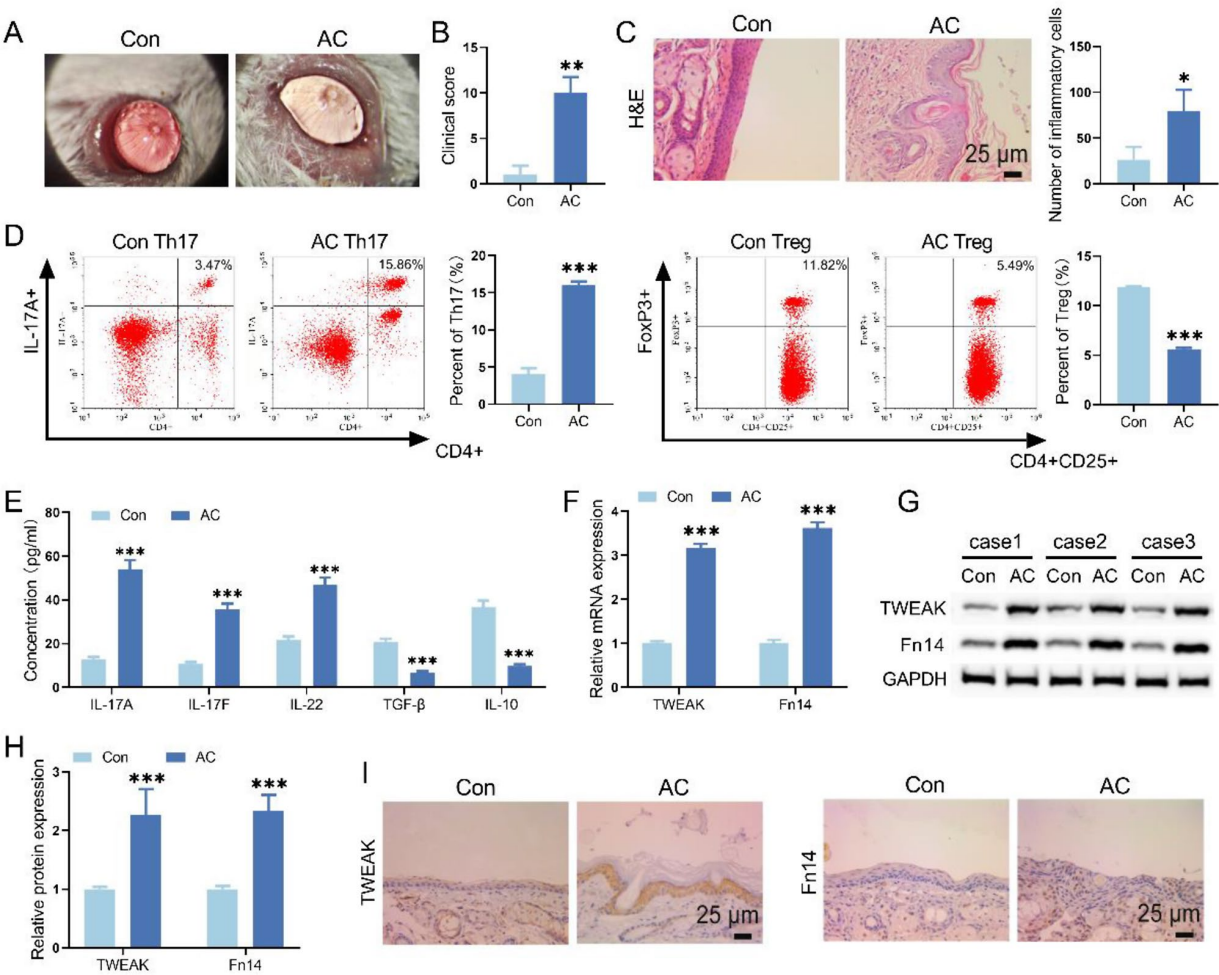
Th17/Treg cell differentiation ratio and TWEAK/Fn14 signaling level in AC mice. (**A**) The state of mice ocular surface was observed by slit-lamp. (**B**) The clinical scores of mice were performed according to the status of eyelid, conjunctiva and cornea. (**C**) HE staining was utilized to evaluate the pathological changes of conjunctival tissue in mice. (**D**) The proportion of Th17 or Treg cells in spleen of mice was assessed by flow cytometry. (**E**) The levels of Th17 and Treg cytokines in mice spleen were evaluated by ELISA. (**F-H**) TWEAK and Fn14 levels in ocular conjunctival tissue were measured by qRT-PCR and WB. (**I**) IHC was used to observe the protein level of TWEAK and Fn14 in ocular conjunctival tissue. ^*^p < 0.05, ^**^p < 0.01, ^***^p < 0.001 vs. Control

Under slit-lamp, AC mice presented conjunctivitis symptoms around the eyes, which were manifested as edema and increased secretion, compared with mice in Control group (Fig. 1A). HE staining implied inflammatory cell infiltration and disordered cell arrangement in AC mice conjunctival tissue, while conjunctival tissue in Control group was normal (Fig. 1C). In addition, the AC clinical score of the AC group was significantly higher than that of the Control group, indicating successful modeling (Fig. 1B). By flow cytometry, it was found that the proportion of Th17 cells significantly increased, while the proportion of Treg cells significantly decreased in the spleen of AC mice (Fig. 1D, S1). Besides, the results of ELISA confirmed that AC caused increased levels of Th17 cytokines IL-17 A, IL-17 F, and IL-22 and decreased levels of Treg cytokines TGF-β and IL-10 (Fig. 1E). Elevated mRNA and protein levels of TWEAK and Fn14 in AC mice were also revealed by qRT-PCR, WB, and IHC (Fig. 1F-I). These findings displayed that altered Th17/Treg cell differentiation balance and TWEAK/Fn14 signaling levels existed in AC mice.

### TWEAK knockdown relieved conjunctivitis of AC mice

**Fig. 2:**
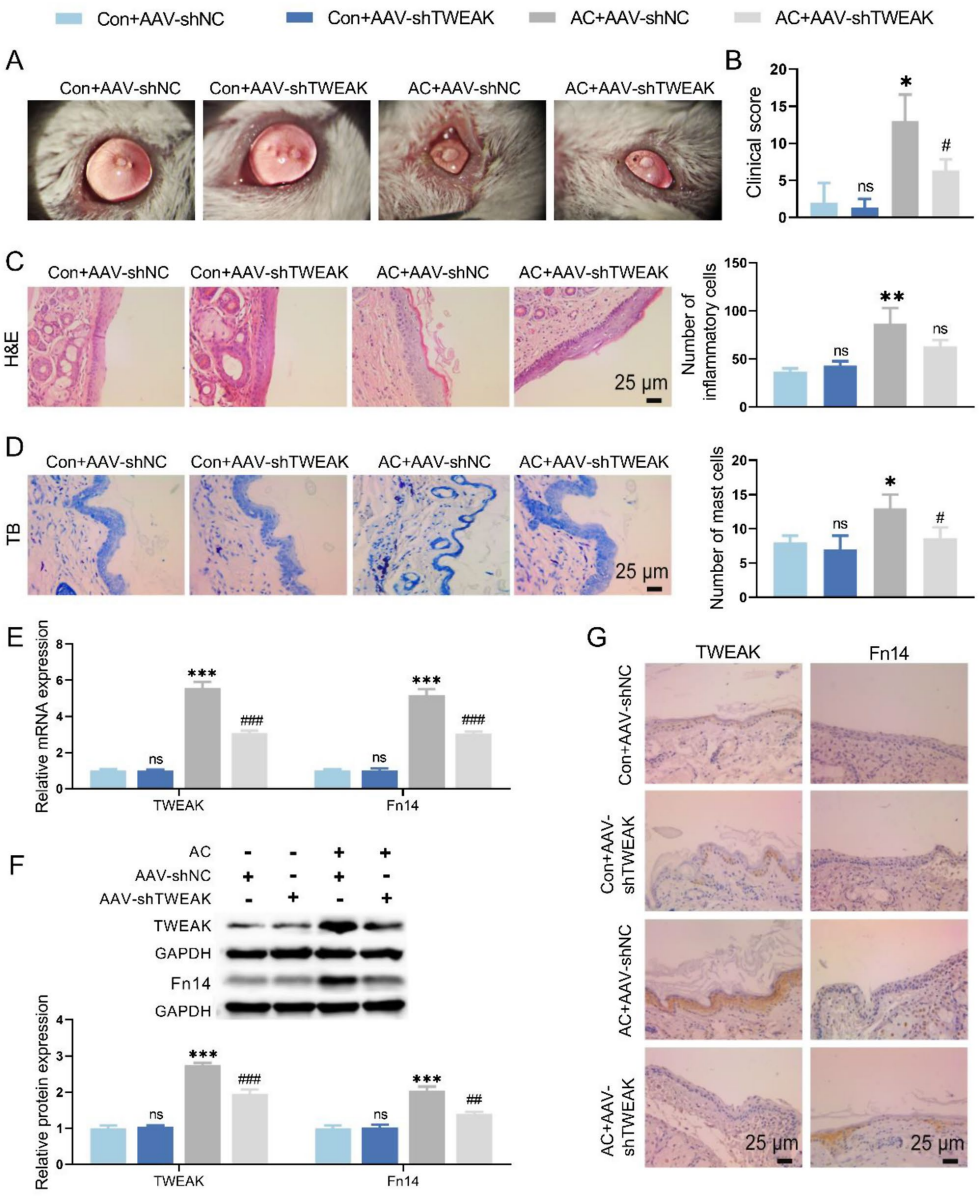
TWEAK knockdown affected conjunctivitis in AC mice. (**A**) The effect of TWEAK knockdown on the state of mice ocular surface was observed under slit-lamp. (**B**) Clinical score of eyelid, conjunctiva and cornea to assess the effect of TWEAK knockdown in AC mice. (**C-D**) HE staining and TB staining were utilized to evaluate the effect of TWEAK knockdown on conjunctivitis in mice. (**E-G**) The levels of TWEAK and Fn14 in conjunctival tissue were detected by qRT-PCR, IHC and WB. ^*^p < 0.05, ^**^p < 0.01, ^***^p < 0.001 vs. Con + AAV-shNC; ^#^*p* < 0.05, ^##^*p* < 0.01, ^###^*p* < 0.001 vs. AC + AAV-shNC; ns, no significant difference

It was observed that TWEAK knockdown reduced conjunctivitis symptoms and clinical scores in AC mice by slit-lamp (Fig. 2A, B). HE staining verified that TWEAK knockdown reduced inflammatory cell infiltration, cell disarrangement and thickening of the conjunctiva in AC mice conjunctiva (Fig. 2C). Moreover, it was confirmed that reduced mast cell and relieved inflammation were observed in conjunctival tissue of AC mice with TWEAK knockdown compared to the AC model group by TB staining (Fig. 2D). The findings of qRT-PCR, WB and IHC assays corroborated that TWEAK knockdown induced reduction of mRNA and protein levels of TWEAK and Fn14 in AC mice (Fig. 2E-G). Thus, both conjunctivitis and TWEAK/Fn14 signaling pathways were suppressed by knockdown of TWEAK in AC mice.

### TWEAK inhibited Treg differentiation and promoted Th17 differentiation

**Fig. 3:**
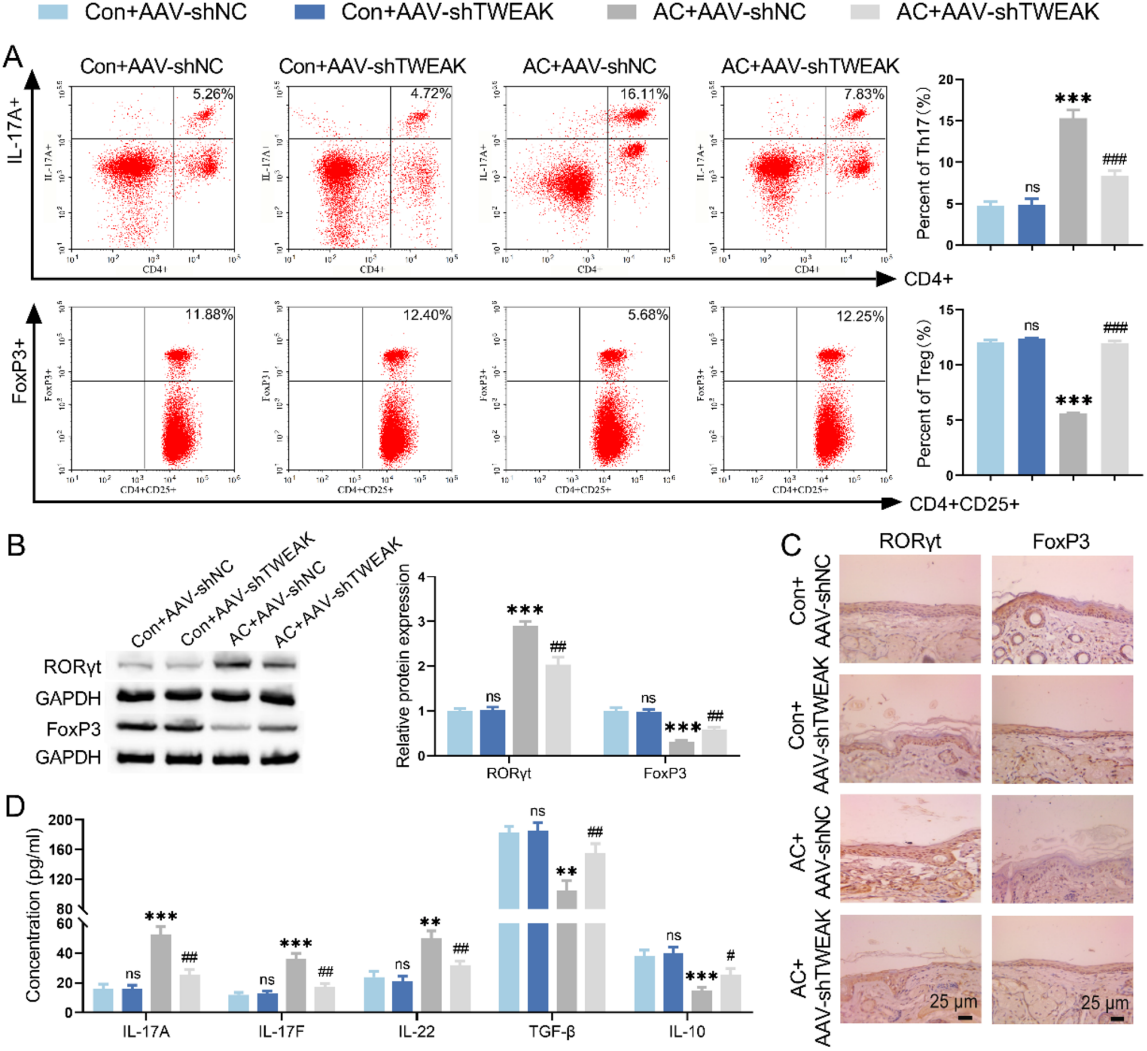
TWEAK regulated the Th17/Treg cell ratio in AC mice. (**A**) The effect of TWEAK knockdown on Th17 and Treg cell ratio in spleen of AC mice was observed by flow cytometry. (**B-C**) WB and IHC were employed to evaluate the expression levels of FoxP3 and RORγt in mice conjunctival tissue. (**D**) The effect of TWEAK knockdown on the levels of Th17 and Treg cytokines in mice spleen were evaluated by ELISA. ^*^p < 0.05, ^**^p < 0.01, ^***^p < 0.001 vs. Con + AAV-shNC; ^#^*p* < 0.05, ^##^*p* < 0.01, ^###^*p* < 0.001 vs. AC + AAV-shNC; ns, no significant difference

The findings of flow cytometry revealed that TWEAK knockdown resulted in a decreased proportion of Th17 cells and an increased proportion of Treg cells in the spleen of AC mice (Fig. 3A, S1). As detected by WB and IHC, the increased level of Treg differentiation transcription factor FoxP3 and decreased level of Th17 differentiation transcription factor RORγt in conjunctival tissue of AC mice were caused by TWEAK knockdown (Fig. 3B-C). Furthermore, compared with the AC model group, the levels of IL-17 A, IL-17 F and IL-22 were reduced, while the levels of TGF-β and IL-10 were increased in the AC mice with silencing TWEAK by ELISA (Fig. 3D). Therefore, silencing TWEAK promoted T cell differentiation into Treg phenotype and inhibited it into Th17 phenotype in AC mice spleen.

### TWEAK knockdown enhanced Nrf2/HO-1 signaling pathway in AC mice

**Fig. 4:**
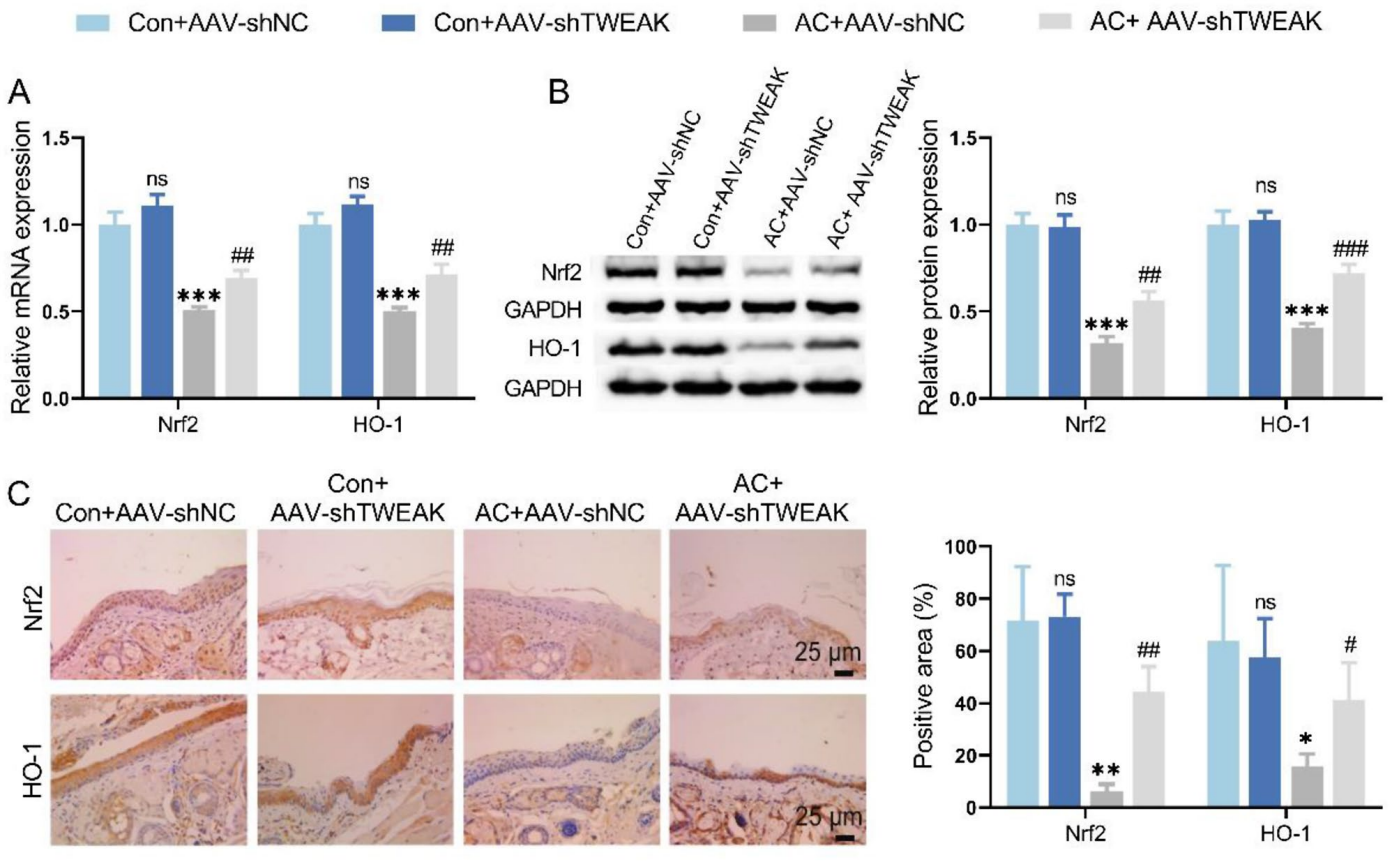
TWEAK knockdown promoted Nrf2/HO-1 signaling pathway in AC mice. (**A**) qRT-PCR was utilized to measure the effect of TWEAK knockdown on Nrf2 and HO-1 mRNA levels in conjunctival tissue of mice. (**B-C**) The effect of TWEAK knockdown on protein levels of Nrf2 and HO-1 in conjunctival tissue of mice were detected by WB and IHC. ^*^p < 0.05, ^**^p < 0.01, ^***^p < 0.001 vs. Con + AAV-shNC; ^#^*p* < 0.05, ^##^*p* < 0.01, ^###^*p* < 0.001 vs. AC + AAV-shNC; ns, no significant difference

The results of qRT-PCR and WB detection demonstrated that the mRNA and protein levels of Nrf2 and HO-1 in AC mice were significantly decreased compared with normal mice. However, TWEAK knockdown presented a restorative effect on Nrf2 and HO-1 levels (Fig. 4A-B). The positive staining area of Nrf2 and HO-1 proteins in the conjunctival tissue of AC mice was significantly smaller than that of normal mice via IHC assay. This positive staining area of AC mice was enlarged by silencing TWEAK (Fig. 4C). In consequence, TWEAK knockdown exerted an enhanced Nrf2/HO-1 signaling pathway in conjunctival tissue of AC mice.

### TWEAK regulated Th17/Treg cell differentiation balance via Nrf2/HO-1

**Fig. 5:**
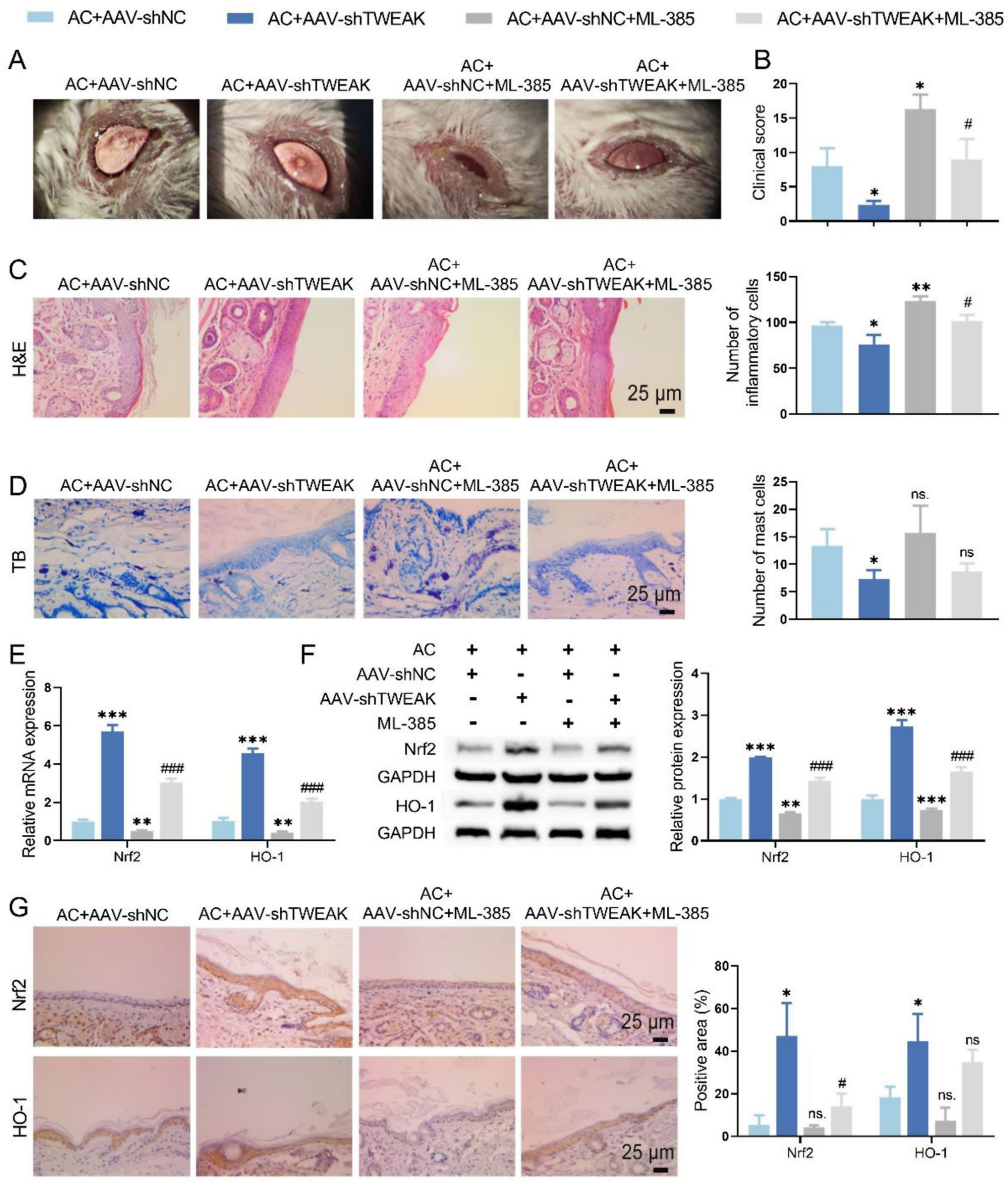
Inhibition of Nrf2/HO-1 signaling pathway affected the improvement of conjunctivitis in AC mice from TWEAK knockdown. (**A**) The effect of Nrf2 inhibitor on ocular surface status was observed by slit-lamp in AC mice with TWEAK knockdown. (**B**) Clinical score of eyelid, conjunctiva and cornea to assess the effect of Nrf2 inhibitor in AC mice with TWEAK knockdown. (**C-D**) HE staining and TB staining were employed to evaluate the effect of Nrf2 inhibitor on conjunctivitis in AC mice with TWEAK knockdown. (**E-G**) The levels of Nrf2 and HO-1 in conjunctival tissue were detected by qRT-PCR, IHC and WB. ^*^p < 0.05, ^**^p < 0.01, ^***^p < 0.001 vs. AC + AAV-shNC; ^#^*p* < 0.05, ^##^*p* < 0.01, ^###^*p* < 0.001 vs. AC + AAV-shTWEAK

Conjunctivitis symptoms and scores increased in AC mice with silencing TWEAK after treatment with Nrf2 inhibitor (Fig. 5A-B). HE and TB staining implied that Nrf2 inhibitor almost eliminated the relief of TWEAK knockdown on inflammation of conjunctival tissue in AC mice (Fig. 5C-D). Additionally, Nrf2 inhibitor reversed the enhancement of the Nrf2/HO-1 signaling pathway in AC mice induced by TWEAK knockdown, as confirmed by qRT-PCR, IHC, and WB assays (Fig. 5E-G).

**Fig. 6:**
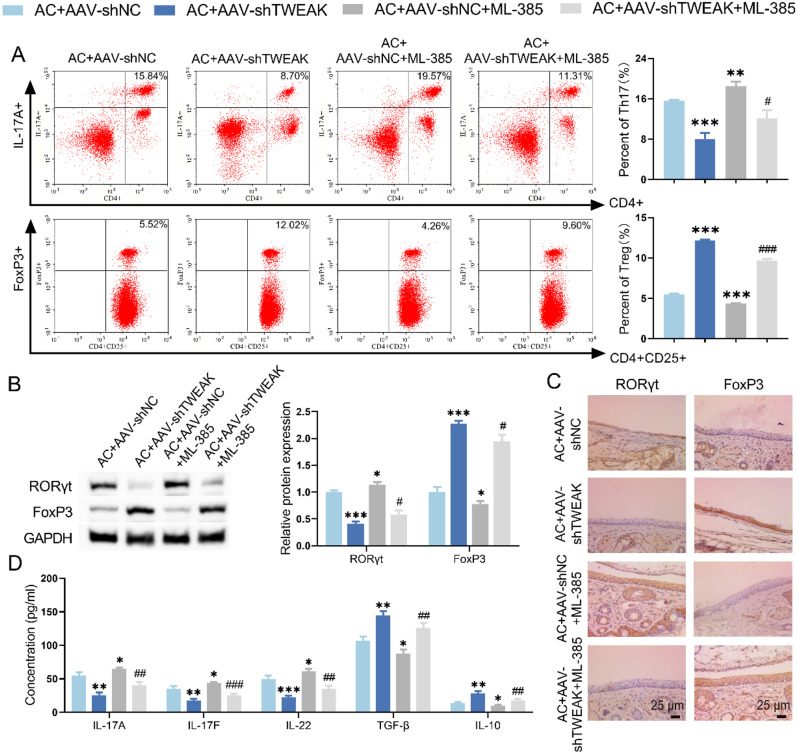
Inhibition of Nrf2/HO-1 signaling pathway affected Th17/Treg cell ratio in AC mice with TWEAK knockdown. (**A**) The effect of Nrf2 inhibitor on Th17/Treg cell ratio in AC mice with TWEAK knockdown was assessed by flow cytometry. (**B-C**) WB and IHC assays were employed to evaluate the expression of FoxP3 and RORγt in conjunctival tissue of AC mice. (**D**) The levels of Th17 and Treg cytokines in mice spleen were detected by ELISA. ^*^p < 0.05, ^**^p < 0.01, ^***^p < 0.001 vs. AC + AAV-shNC; ^#^*p* < 0.05, ^##^*p* < 0.01, ^###^*p* < 0.001 vs. AC + AAV-shTWEAK

The results of flow cytometry corroborated that Nrf2 inhibitor increased the proportion of Th17 cells and decreased the proportion of Treg cells in the spleen of AC mice with silencing TWEAK (Fig. 6A, S1). Besides, Nrf2 inhibitor negated the effect of TWEAK knockdown on the expression levels of FoxP3 and RORγt, which were detected by WB and IHC (Fig. 6B-C). The ELISA detection confirmed that Nrf2 inhibitor upregulated the level of IL-17 A, IL-17 F and IL-22 and downregulated the level of TGF-β and IL-10 in AC mice with TWEAK knockdown (Fig. 6D). In summary, TWEAK knockdown induced the remission of conjunctivitis and the differentiation of T cells to Treg phenotype in AC mice, which was counteracted by Nrf2 inhibitor. Therefore, TWEAK/Fn14 promoted Th17/Treg imbalance by inhibiting the Nrf2/HO-1 pathway, contributing to the development of conjunctivitis in AC mice.

## Discussion

In recent years, several researchers have observed a marked increase in the prevalence of allergic diseases, including different forms of AC. The occurrence of AC has adverse effects on daily life and work of people (Bilkhu et al. 2012; Pawankar 2014). In this work, the effects of TWEAK/Fn14 on conjunctivitis symptoms and Th17/Treg cell balance in AC mouse models were observed and evaluated. The specific mechanism of TWEAK/Fn14 inhibiting Nrf2/HO-1 signaling pathway to regulate Th17/Treg cell balance during AC progression was elucidated.

TWEAK, also known as TNFSF12, is a multifunctional cytokine that regulates many cellular activities, including proliferation, migration, differentiation, apoptosis, angiogenesis, and inflammation (Hu et al. 2017; Winkles 2008). Moreover, the interaction of TWEAK with Fn14 receptors stimulates various cellular responses and activates various signaling pathways, which are involved in the pathogenesis of cerebral ischemia, chronic inflammatory diseases and cancer (Blanco-Colio 2014; Cheng et al. 2013; Ratajczak et al. 2022). Existing literatures have confirmed that the enhancement of TWEAK/Fn14 signaling induces the secretion of pro-inflammatory cytokines and promotes the occurrence of inflammation (Campbell et al. 2004; Harada et al. 2002; Wang et al. 2017). The experimental data of this study corroborated that the levels of TWEAK and Fn14 were significantly upregulated under AC. Additionally, TWEAK knockdown inhibited TWEAK/Fn14 signaling and relieved conjunctivitis symptoms of AC mice. These results confirmed that TWEAK/Fn14 signaling levels were elevated in AC state and that it promoted inflammation in AC mice, which is consistent with the literature. Although TWEAK/Fn14 activation has been reported to be involved in the occurrence of multiple skin inflammation, this is the first time it has been explored in a model of AC disease (Liu et al. 2017).

The dysregulation of Th17/Treg balance plays a role in inflammatory diseases of the lungs and intestines and chronic inflammation associated with obesity and metabolic diseases (Thomas et al. 2023; Yan et al. 2020; Zhang et al. 2021b). Furthermore, La Rosa et al. clarified that Th17 and Treg are contributors to the pathogenesis of conjunctivitis (La Rosa et al. 2013). Flow cytometry analysis revealed an elevated proportion of Th17 cells and a decreased proportion of Treg cells in our AC mouse model, indicative of an imbalance in the Th17/Treg ratio. Besides, the transcription factor RORγt drives the differentiation of naive CD4 T cells into Th17 cells (Ivanov et al. 2006). The transcription factor FoxP3 promotes its differentiation into Treg cells (Kanamori et al. 2016). In this investigation, the RORγt expression level was significantly higher in AC mice compared to normal mice, whereas the FoxP3 expression level was significantly lower in AC mice. Thus, in AC state, Th17 cell differentiation was promoted, while Treg cell differentiation was inhibited.

Th17 and Treg cells exhibit contrasting functions in inflammation and immune response (Littman and Rudensky 2010). On the one hand, Th17 cells secrete IL-17, IL-22, and IL-23 to recruit neutrophils, leading to aggravated inflammation at the site of infection. On the other hand, Treg cells generate the anti-inflammatory cytokines IL-10 and TGF-β, which inhibit the activity of a variety of immune cells to suppress the immune response. The study demonstrated that the upregulation of RORγt, IL-17 A, IL-17 F, and IL-22 during conjunctival inflammation in AC mice was observed. However, when TWEAK knockdown resulted in a remission of conjunctival inflammation, the upregulated cytokines in AC mice were replaced with changed to FoxP3, IL-10, and TGF-β. Therefore, TWEAK regulated the Th17/Treg balance to favor the proinflammatory Th17 phenotype, contributing to the progression of conjunctivitis in AC mice. In addition, it has been reported that the levels of IL-17 A, IL-17 F and RORγt in conjunctival tissue of experimental AC mice are increased, while the levels of FoxP3 and IL-10 are inhibited (Chen et al. 2019). The IL-10 exhibits a lower expression in tears of patients with perennial AC (Salazar et al. 2019). The evidence of these research is consistent with our results.

The Nrf2/HO-1 signaling pathway plays an anti-inflammatory and antioxidant role in many disease processes (Mazur-Bialy and Pocheć 2021; Wang et al. 2021). In a study of acute lung injury, elevated Fn14 expression levels and increased oxidative stress and inflammatory responses are observed, limiting the activity of the Nrf2/HO-1 signaling pathway to exacerbate inflammation and tissue damage (Guan et al. 2022). The present study displayed that Nrf2/HO-1 signaling level was inhibited in the AC state, which was mitigated by silencing TWEAK. Therefore, TWEAK/Fn14 inhibited the activation of Nrf2/HO-1 signaling pathway, which was consistent with the results in the literature. Furthermore, existing studies have confirmed that regulating Th17/Treg balance and enhancing Nrf2/HO-1 pathway signaling contribute to improvement of allergic rhinitis (Ma et al. 2023; Van Nguyen et al. 2020). However, the interaction between Nrf2/HO-1 pathway and Th17/Treg balance in allergic rhinitis has not been elucidated. In this work, blocking the Nrf2/HO-1 pathway with an ML-385 inhibitor reversed the direction of TWEAK regulating Th17/Treg balance in AC mice. As a result, TWEAK/Fn14 promoted Th17/Treg imbalance by inhibiting the Nrf2/HO-1 pathway, which leads to the development of inflammation.

TWEAK/Fn14 is known to regulate cellular activity primarily through the activation of TRAF and NF-κB signaling pathways (Cheng et al. 2013; Hu et al. 2017). Research has demonstrated that modulating the Nrf2/HO-1 pathway and suppressing NF-κB/IκB activation ameliorate nasal inflammation in mice (Van Nguyen et al. 2020). Additionally, TWEAK/Fn14 has been found to induce the release of pro-inflammatory cytokines in hepatic stellate cells via the NF-κB/STAT3 pathway (Wang et al. 2017). Further investigation is warranted to determine whether TWEAK/Fn14 activates the NF-κB signaling pathway while inhibiting Nrf2/HO-1 signaling in AC model mice.

In this study, the AC mouse models were utilized to investigate the involvement of TWEAK/Fn14 in the pathogenesis of AC, marking the first instance of such exploration. Furthermore, the impact of the Nrf2/HO-1 pathway on the balance of Th17/Treg cells in AC was further explored. The findings implied that TWEAK/Fn14 contributed to the imbalance of Th17/Treg cells in AC mice by inhibiting the Nrf2/HO-1 signaling pathway, although the precise mechanism remained uncertain. Additionally, TWEAK might influence other signaling pathways in AC, such as the NF-κB pathway. The results of this study provided new insights for exploring new therapeutic targets for AC.

## Conclusion

In summary, this study elucidated the regulatory effect of TWEAK/Fn14 on conjunctivitis and Th17/Treg balance in AC mice using OVA-induced AC models for the first time. TWEAK/Fn14 caused Th17/Treg imbalance by inhibiting Nrf2/HO-1 pathway, which might be one potential mechanism of the exacerbation of AC.

## Electronic supplementary material

Below is the link to the electronic supplementary material.

Supplementary Material 1

## Data Availability

All data generated and/or analyzed during the current study are available from the corresponding author on reasonable request.

## Abbreviations

AAV: Adeno-associated virus
AC: Allergic conjunctivitis
BCA: Bicinchoninic acid
BSA: Bovine serum albumin
DAPI: 4’,6-diamidino-2-phenylindole
ECL: Enhanced chemiluminescence
ELISA: Enzyme-linked immunosorbent assay
Fn14: Factor-inducible 14
HE: Hematoxylin-eosin
HO-1: Heme oxygenase-1
HRP: Horseradish peroxidase
IHC: Immunohistochemical
IL-10: Interleukin-10
IL-17A: Interleukin-17 A
IL-17F: Interleukin-17 F
IL-22: Interleukin-22
Nrf2: Nuclear factor erythroid-2 related factor 2
OVA: Ovalbumin
PVDF: Polyvinylidene fluoride
qRT-PCR: Quantitative real-time PCR
TB: Toluidine Blue
TGF-β: Transforming growth factor-beta
Th17: T helper 17
TNF: Tumor necrosis factor
Treg: Regulatory T
TWEAK: Tumor necrosis factor-like weak inducer of apoptosis
WB: Western blotting
