# Supplementary material for: TWEAK/Fn14 disrupts Th17/Treg balance and aggravates conjunctivitis by inhibiting the Nrf2/HO-1 pathway in allergic conjunctivitis mice
Source: Mol Med. 2024 Nov 26;30:233. doi: 10.1186/s10020-024-01004-5 (PMC11590473; doi:10.1186/s10020-024-01004-5)

Figure 1G


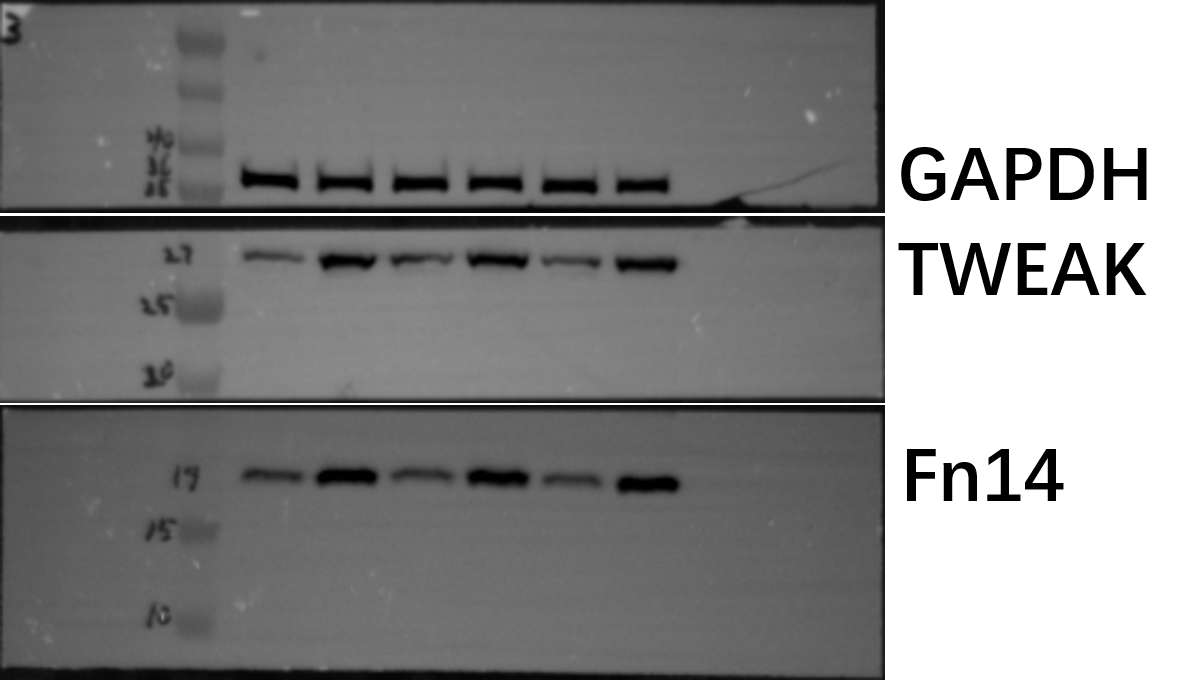


Figure 1G- TWEAK Figure 1G- Fn14 Figure 1G- GAPDH


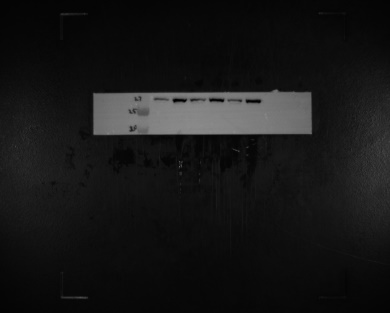

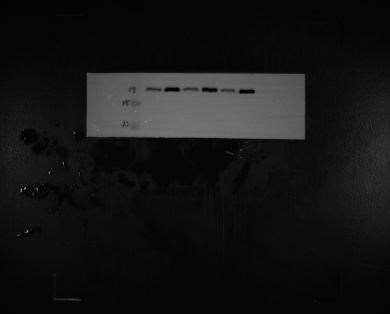

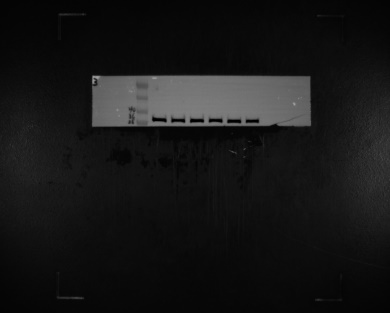


Figure 2F- Fn14


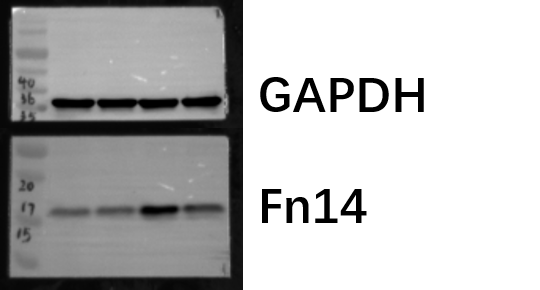


Figure 2F- Fn14 Figure 2F- GAPDH (Fn14)


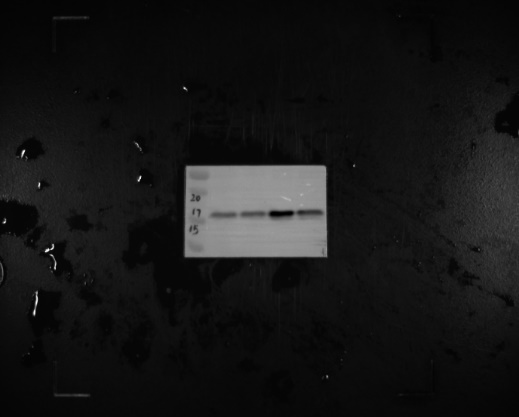

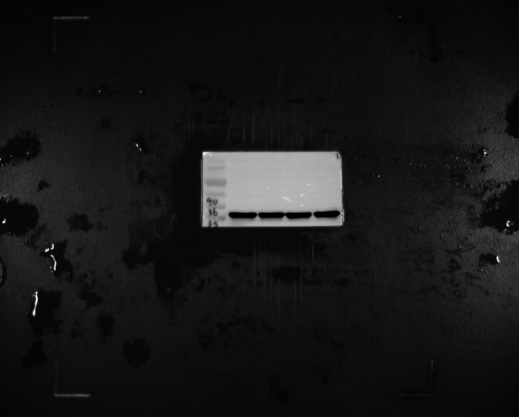


Figure 2F- TWEAK


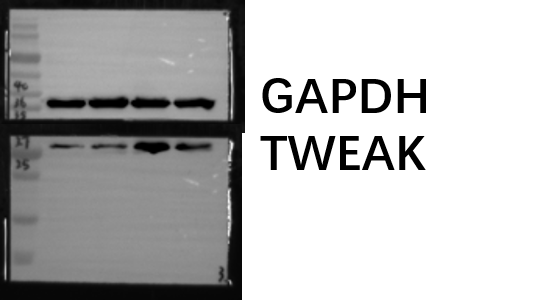


Figure 2F- TWEAK Figure 2F- GAPDH (TWEAK)


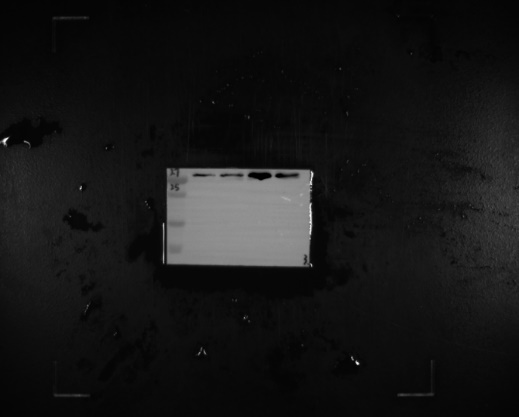

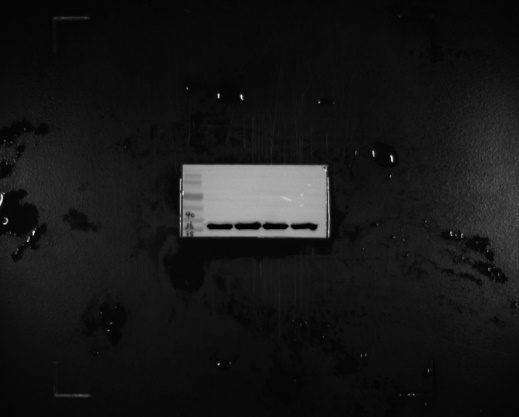


Figure 3B- RORγt


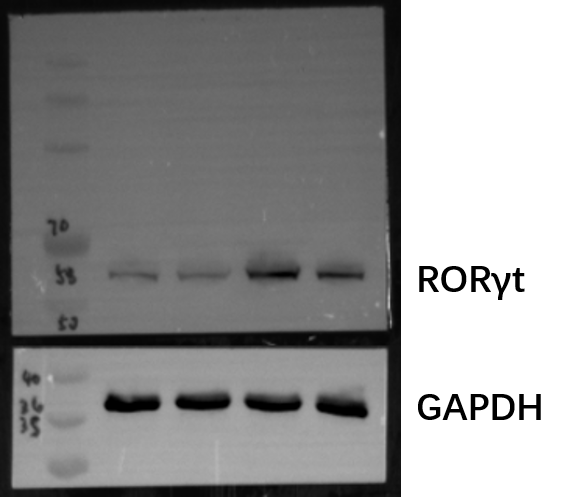


Figure 3B- RORγt Figure 3B- GAPDH (RORγt)


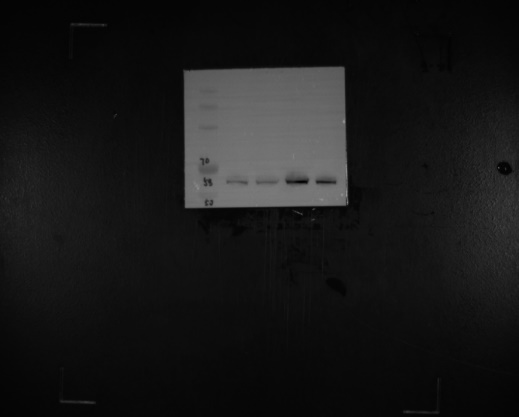

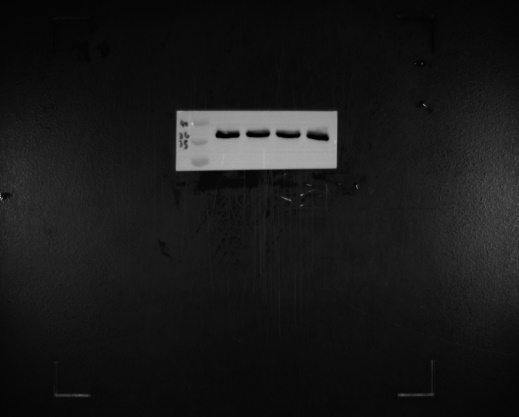


Figure 3B- FoxP3


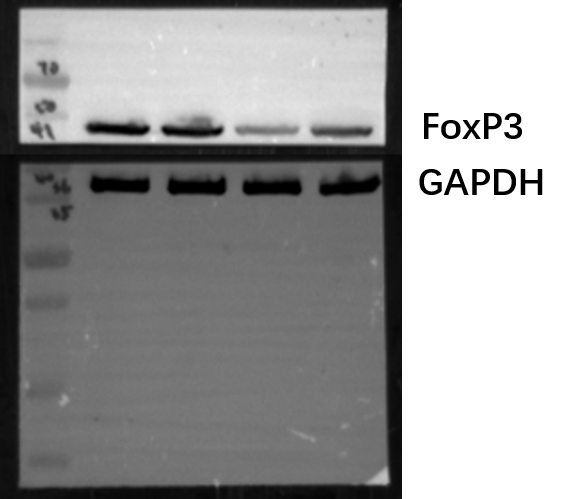


Figure 3B- FoxP3 Figure 3B- GAPDH (FoxP3)


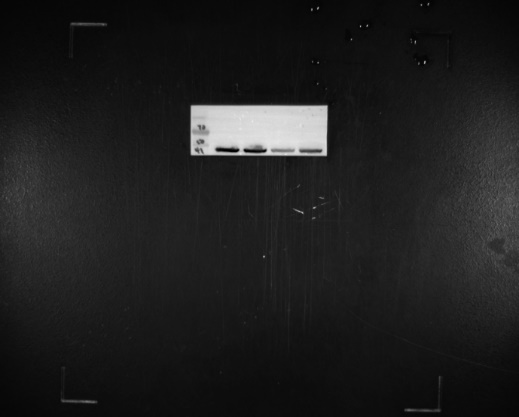

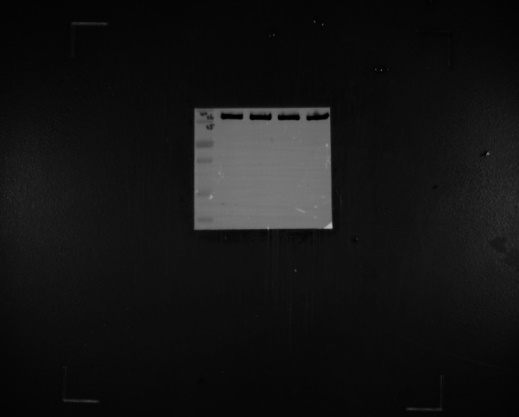


Figure 4B- Nrf2


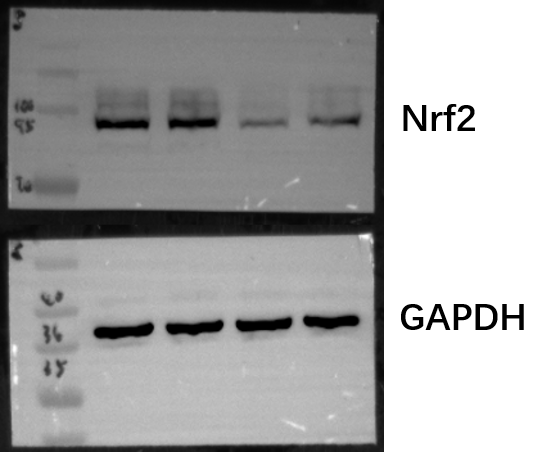


Figure 4B- Nrf2 Figure 4B- GAPDH (Nrf2)


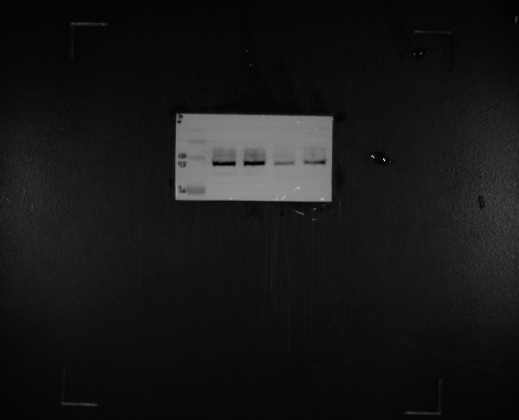

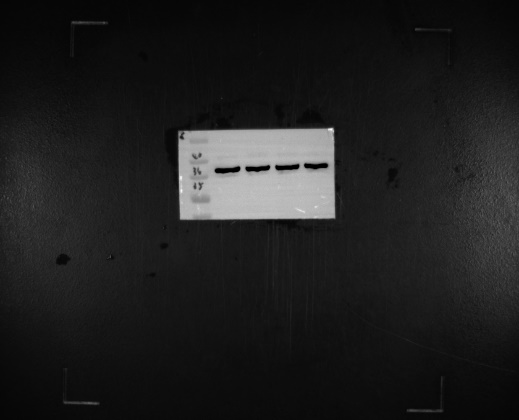


Figure 4B- HO-1


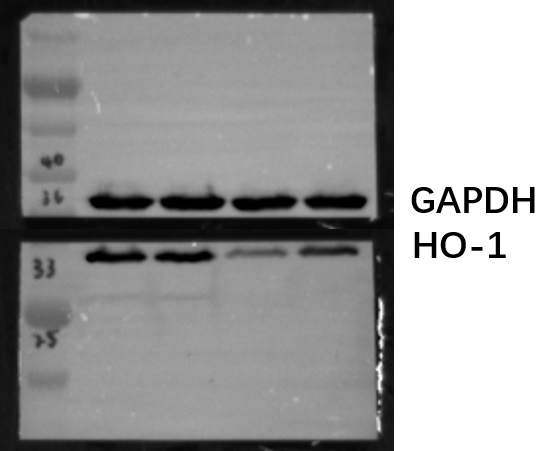


Figure 4B- HO-1 Figure 4B- GAPDH (HO-1)


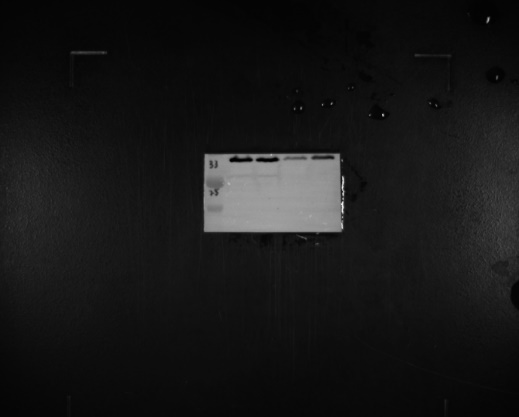

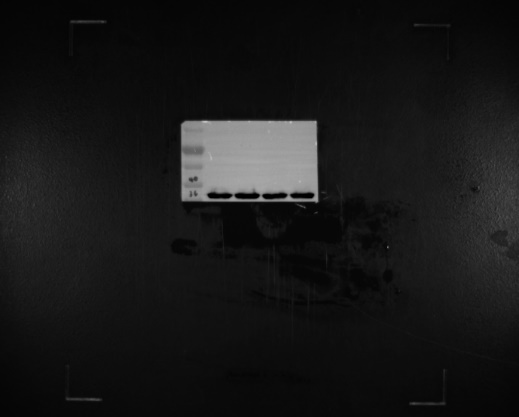


Figure 5F- Nrf2


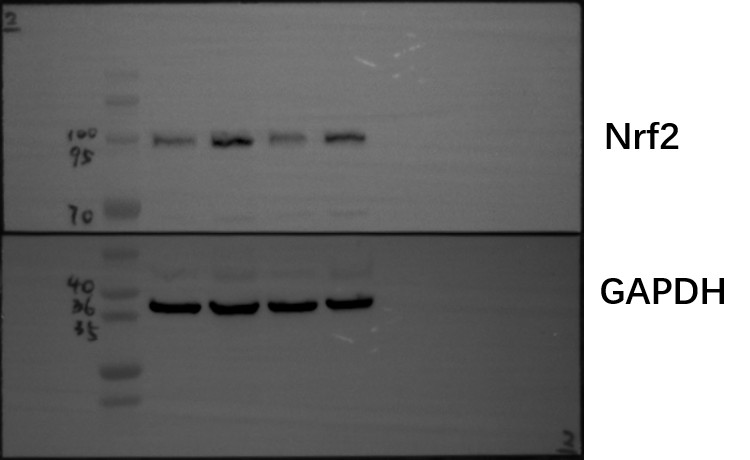


Figure 5F- Nrf2 Figure 5F- GAPDH (Nrf2)


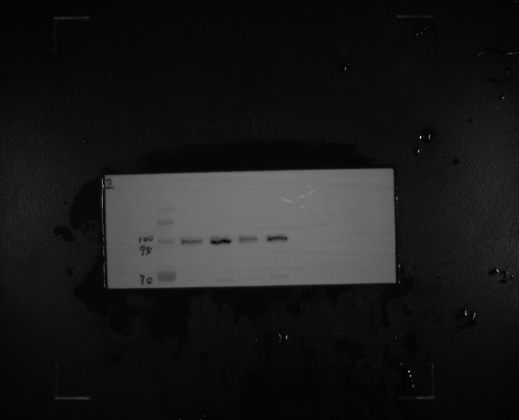

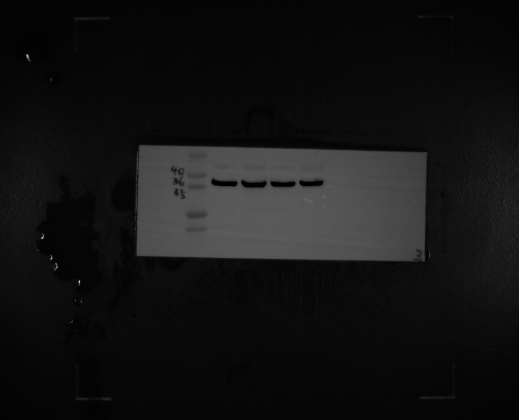


Figure 5F- HO-1


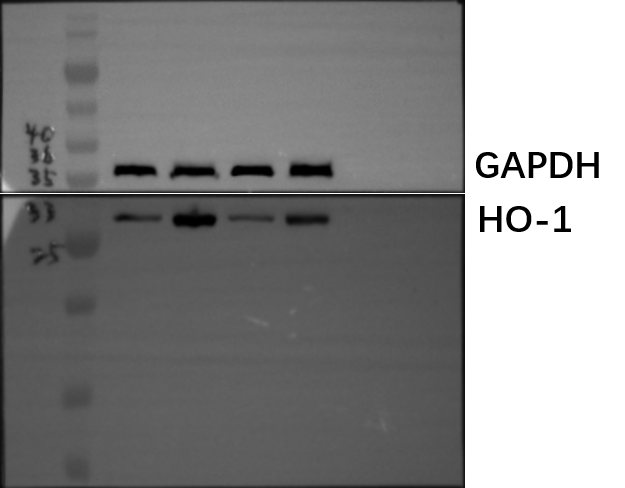


Figure 5F- HO-1 Figure 5G- GAPDH (HO-1)


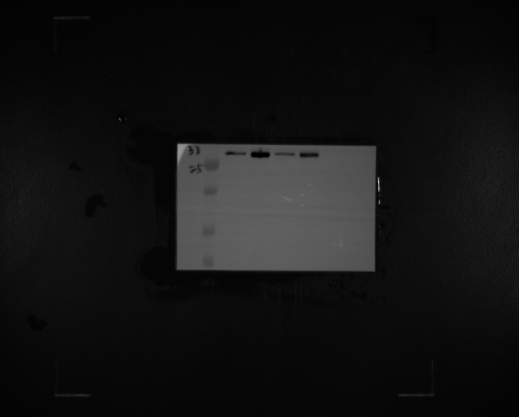

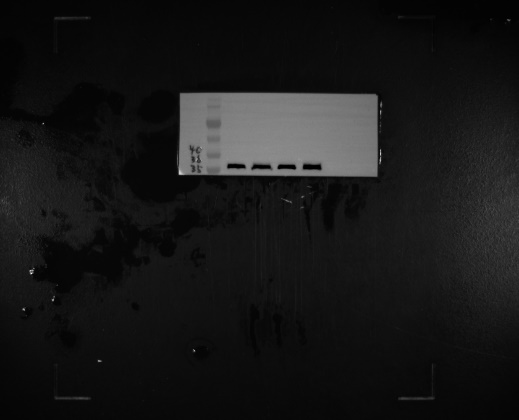


Figure 6B


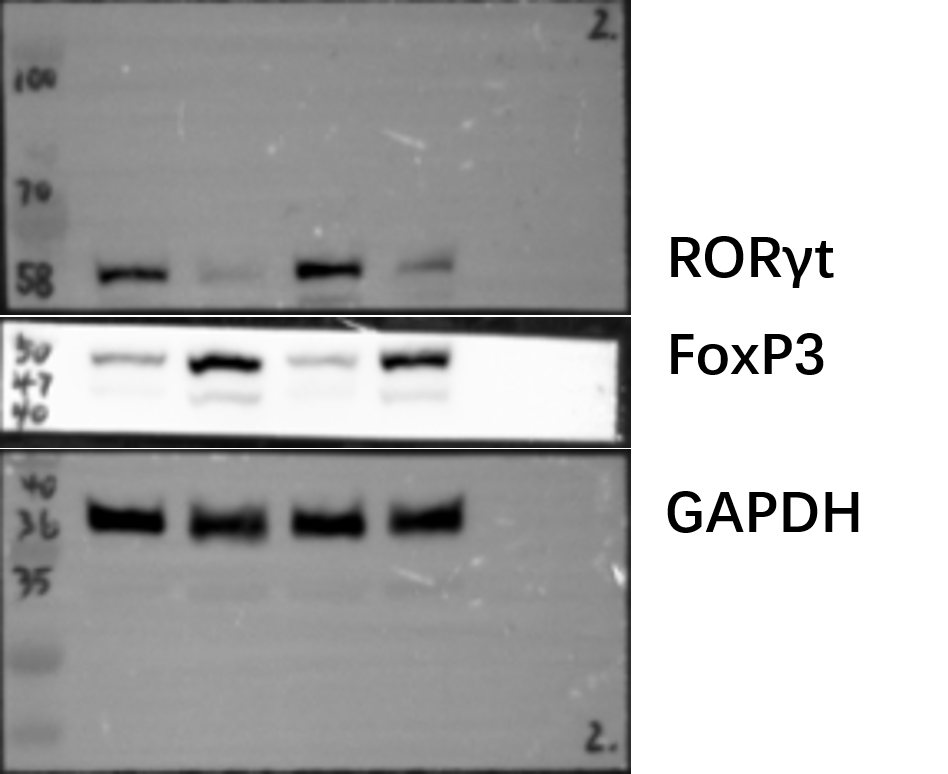


Figure 6B- RORγt Figure 6B- FoxP3 Figure 6B- GAPDH


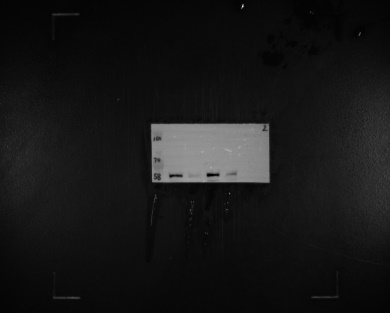

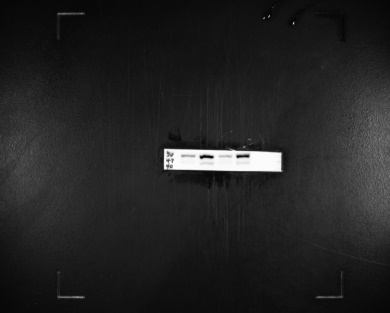

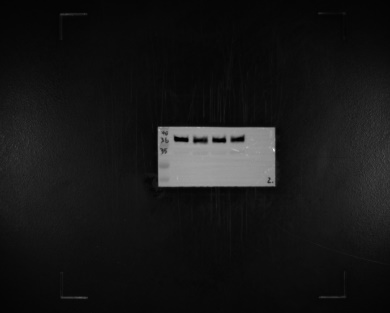

Supplement: Supplementary file 1 — Supplementary Material 1 [file 10020_2024_1004_MOESM1_ESM.docx]
